# Advances in the Application of Aerobic Granular Sludge for the Removal of Emerging Contaminants in Municipal and Industrial Wastewater

**DOI:** 10.3390/molecules30173522

**Published:** 2025-08-28

**Authors:** Gobi Kanadasan, Choon Aun Ng, Vel Murugan Vadivelu, Mohammad J. K. Bashir

**Affiliations:** 1 Department of Chemical Engineering, Universiti Tunku Abdul Rahman, Kampar 31900, Perak, Malaysia; 2 Department of Environmental Engineering, Universiti Tunku Abdul Rahman, Kampar 31900, Perak, Malaysia; ngca@utar.edu.my; 3 School of Chemical Engineering, Universiti Sains Malaysia, Engineering Campus, Nibong Tebal 14300, Penang, Malaysia; chvel@usm.my; 4 School of Engineering and Technology, Central Queensland University, 120 Spencer St., Melbourne, VIC 3000, Australia

**Keywords:** aerobic granules, emerging contaminants, mechanism, treatment performance

## Abstract

Aerobic granules are dense three-dimensional microbial aggregates which are known for their excellent settling ability, high biomass retention, and simultaneous biological reaction due to their multilayered structure. All these features enable the aerobic granules to remove emerging contaminants, such as pharmaceutical and personal care products (PPCPs), endocrine-disrupting compounds (EDCs), microplastics, and per- and polyfluoroalkyl substances (PFASs) in municipal and industrial wastewater. This review discusses the development and application of the aerobic granules, especially in a sequencing batch reactor (SBR) with a height over diameter (H/D) ratio of 5 to 10. The mechanisms of EC removal in aerobic granules and the removal efficiency of the ECs by aerobic granules were also scrutinized, with the reported removal efficiency ranging from 10–100% for PPCPs, 84–94% for EDCs, 74–95% for microplastics, and more than 85% for PFAs. In spite of the huge potential of aerobic granular technology, its large-scale implementation is hampered by operational and scaling challenges. Future research should focus on optimizing the operational parameters and overcoming the scale-up barrier to fully leverage the potential of aerobic granules in removing ECs.

## 1. Introduction

The emergence of new pollutants in wastewater has caused serious concerns in recent years. Pollutants, such as per- and polyfluoroalkyl substances (PFASs), pharmaceutical waste, personal care products, nanomaterials, and microplastics, have been detected frequently in wastewater in the past 10 years and are collectively termed as emerging contaminants (ECs) [1]. These pollutants have exhibited the possibility of bioaccumulation in the ecosystem and human body. The adverse effect of these pollutants on humankind have sparked the need to remove them before they can enter the food chain.

The growing population and demand for products, such as cleaning products, shampoo, eye makeup, nail polish, water-resistant fabrics, like those used in jackets, and non-stick cookware, are the major sources of these emerging pollutants. The source point for the ECs can be categorized as direct and indirect. Direct sources of ECs include the direct release of waste, such as shampoo and cleaning products, into water bodies [2]. Meanwhile, indirect sources of ECs include agricultural runoffs, leaching from landfill, and urban runoff [3]. As a consequence of these sources, ECs can be commonly found in municipal wastewater and industrial wastewater. Commonly, biological treatments are used to remove these types of contaminants from wastewater sources. 

However, the existing biological wastewater treatment is not designed to treat ECs. Currently, the activated sludge system has been predominantly used to treat any incoming wastewater biologically. Unfortunately, the activated sludge cannot biodegrade or remove the emerging contaminants from the wastewater [4]. ECs could become toxic to the activated sludge in the existing biological treatment system. Therefore, there is an urgent need to use other methods to remove ECs from wastewater.

In tandem with this need, the emergence of aerobic granules as a substitute for activated sludge has been seen in recent times. Aerobic granules are superior to activated sludge in many aspects, such as in terms of their settling ability, biomass per volume ratio, compactness, toxic resistance, and also their simultaneous nutrient removal from wastewater [5,6,7]. Realizing the superior ability of the aerobic granules, a body of research has been conducted to investigate the ability of aerobic granules to remove ECs. The removal of ECs via aerobic granules was discovered to be happening via several different methods, namely biodegradation, entrapment onto their complex matrix, and adsorption through extracellular polymeric substances (EPSs). However, the full-scale implementation of aerobic granular technology for the removal of ECs is hampered by several factors. Primarily, the granule formation and stability are concerning when implementing this technology at full-scale. Apart from that, the impact of the emerging contaminants towards the microbial community of the aerobic granules is not fully discovered, which impairs the scale up of this technology. The operational parameters for the transformation from lab-scale to full-scale is yet to be established as well, which again hampers the transformation from lab-scale to full-scale. Given these challenges, there is a critical need to investigate how aerobic granules interact with emerging contaminants under variable conditions, and to identify the operational factors that govern their stability and efficiency. This study is, thus, essential to bridge this knowledge gap and support the practical implementation of aerobic granular systems for EC removal.

This review aims to comprehensively scrutinize the latest advances in applying aerobic granular technology to remove ECs, while discovering the bottlenecks. First, an overview of aerobic granular technology is be discussed, as well as an overview of ECs and their environmental impacts. This is followed by a discussion of the fundamental principles of aerobic granular technology and the reactor configurations used to cultivate aerobic granules. Thereafter, the mechanism involved in the removal of ECs, the removal efficiency of ECs, and the impact of ECs towards the granules’ structure, are critically analyzed. Finally, the challenges present to transform the lab-scale aerobic granular technology to industrial-scale are extensively discussed. The potential future direction of the aerobic granules and their successful implementation to remove ECs is also discussed. 

## 2. Aerobic Granular Sludge Technology

Aerobic granular sludge technology is widely used in wastewater treatment under aerobic conditions. Aerobic granules are agglomerates of activated sludge, taking the form of dense, compact, and layered spherical shapes [8]. They originate from the activated sludge and undergo several transformations in multiple stages before they turn into aerobic granules. Matured aerobic granules are uniform in appearance. Aerobic granules are usually darker in colour compared to the activated sludge [8,9]. The agglomeration process makes the aerobic granules multilayered, and the diversity of the bacterial community can be observed along the layers [7]. As the aerobic granules are compact and spherical, they are highly settleable and can occupy a higher number of microorganisms per unit reactor system. The application of the aerobic granular treatment system occupies only 25% of the space required for conventional wastewater treatment [10]. In addition to that, the need for a clarifier can be eliminated with the application of aerobic granular technology [11,12]. 

The activated sludge presents loosely in its original state. The transformation of activated sludge into aerobic granules is possible within several conducive environments. Usually, the activated sludge is transformed using a sequencing batch reactor (SBR). To transform the activated sludge into aerobic granules, the feeding system of the reactor has to ensure that the feast–famine region can be established in the reactor [13]. Generally, the famine phase is longer compared to the feast phase. During the prolonged famine phase, the activated sludge undergoes endogenous respiration, where energy is derived from the breakdown of internal cell material for basic cellular functions [14]. In the case of the prolonged famine phase, the activated sludge counteracts the condition by releasing a higher amount of extracellular polymeric substance (EPS) during the famine phase. The increased quantity of EPS encapsulates the activated sludge during the famine period. This is a natural strategy for the activated sludge to protect itself from lysis during adverse conditions, such as famine periods. The produced EPS encapsulates the activated sludge, preventing it from being exposed to the adverse conditions surrounding the activated sludge [15]. At the same time, the EPS produced by the activated sludge functions to bind the activated sludge together. The binding process of the activated sludge is the initiation stage of the aerobic granules’ formation [16]. Several researchers have noticed the increase in EPS production upon the introduction of a feast–famine feeding regime [17,18,19]. This naturally paves the way for the binding of the activated sludge and, consequently, the formation of aerobic granules. 

Aerobic granules have proven to be a better choice for wastewater treatment compared to activated sludge for several reasons, as highlighted in Table 1. All the characteristics exhibited by the aerobic granules (as summarized in Table 1) suggest that the aerobic granules are a better alternative for activated sludge usage in wastewater treatment. One of the key characteristics of aerobic granules is their ability to adapt to the environment quicker compared to activated sludge. This characteristic is significant in tackling new pollutants at present and in the future. 

## 3. Emerging Contaminants Targeted

### 3.1. Pharmaceuticals and Personal Care Products (PPCPs)

Pharmaceutical and personal care products (PPCPs) have been gaining attention in recent years due to an increase in their usage. PPCPs include a wide range of substances, such as antibiotics (e.g., erythromycin, ciprofloxacin), pain relievers (e.g., acetaminophen, ibuprofen), antidepressants (e.g., carbamazepine), hormones, soaps, lipsticks, moisturizers, insect repellent, triclosan, and parabens. These products have become integral part of daily human life across most parts of the world. 

The growing presence of PPCPs has been attributed to several factors. In the case of pharmaceutical products, the global demand for their use has surged in recent years due to the emergence of new bacterial and viral infections. In the Asia–Pacific region, countries, like Malaysia, Indonesia, Thailand, and Vietnam, have recorded a notable increase in pharmaceutical consumption over the past decade. Specifically, the compound annual growth rate (CAGR) of the pharmaceutical market in Malaysia, Indonesia, Thailand, and Vietnam has shown a growth of 4.46%, 7.7%, 3.34%, and 3.83%, respectively [27,28,29,30]. These growth rates significantly reflect the significant upward trend in the use of pharmaceutical products.

The growth rate of the pharmaceutical market is largely driven by a rise in antimicrobial resistance due to evolving microorganisms, which necessitates the development of improved or new treatments [31]. Additionally, increased health awareness and self-medication practices among the public have vastly increased the usage of pharmaceutical products. The growing emphasis on personal grooming has paved the way for the high demand for these products. Furthermore, aging societies in certain countries, such as Thailand and Japan, have also escalated the demand for both pharmaceutical and personal care products [32]. 

In tandem with the growth of the pharmaceutical market, the post-consumption release of PPCPs into the environment has been steadily growing since 2000. The incomplete removal of PPCPs in conventional treatment plants, and the direct release of PPCPs from various sources, have further exacerbated the increase in PPCPs in the environment [33,34,35]. The issue of the release of PPCPs into the environment has gained traction due to their persistence and potentially harmful nature. It has been reported that the average amount of PPCPs detected in the effluent of a wastewater treatment plant was 7685.9 ng/L [36]. 

In municipal wastewater, the average concentration of PPCPs detected was between 133–139 μg/L [37]. Although PPCPs were detected in a small amount in the surface water and wastewater initially, the negative consequential effect of PPCPs has invoked interest in removing this component from waste bodies. PPCPs have the ability to induce physiological effects on both human and aquatic life, even at small dosage [38]. As PPCPs are released into water streams, they have direct impact towards the aquatic livings too. The release of PPCPs into water streams exerts selective pressure on microorganisms, leading to the emergence of antibiotic-resistant bacteria in water streams. These bacteria could persist in aquatic environment and potentially enter the human food chain via seafood consumption. Consequently, hard-to-treat infections resulting from these antibiotic-resistant bacteria may arise in humans, posing serious risks to food security [39].

PPCPs commonly detected in the surface water include diclofenac, carbamazepine, triclosan, phthalate, and tramadol [40]. The detection of these PPCPs is primarily due to their ineffective removal by the existing treatment system. The existing treatment plants are poorly equipped to treat these PPCPs, as the existing methods are not flexible enough to remove these emerging contaminants [1]. The release of PPCPs is possible in two ways, i.e., direct and indirect. Direct release of PPCPs originates from pharmaceutical industry wastewater, hospital wastewater, and also cosmetic industry wastewater [41]. Meanwhile, the indirect release of PPCPs comes from domestic wastewater, where they are secreted as a result of the incomplete metabolization of the drugs by humans [42]. 

### 3.2. Endocrine-Disrupting Compounds (EDCs)

A healthy endocrine system in human beings is essential for ensuring a healthy lifestyle. The endocrine system functions to produce, store, regulate, and secrete hormones in the human body [43]. The hormone secretion network must be free from external factors for the human body to function optimally. However, endocrine-disrupting compounds (EDCs) are ever-present around us. EDCs are chemical compounds which disrupts the normal function of the endocrine system in the human body. They are present in the air, water, food sources, and personal care products. These EDCs can penetrate our body through various ways, such as food, air inhalation, water consumption, and through our skin. Disruption to the normal functioning of the endocrine system has been found to affect normal human body functions in many ways. The reproductive system, digestive system, immune system, cardiovascular system, and growth hormones have been found to be affected by EDCs [44]. 

Many EDCs have been reported as being detected in the environment and, eventually, in the human body. EDCs, such as bisphenol A, polychlorinated biphenyls (PCBs), dichlorodiphenyltrichlorethane (DDT), perchlorate, and phthalates, are among the species which have been detected in recent years in water resources [45]. In municipal wastewater, the concentration of the EDCs averaged between 0.4 and 450 ng/L [46]. In a recent finding, it was found that EDCs leached out from food containers upon filling up the containers with food at temperatures between 40 to 100 °C [47]. The leached-out EDCs enter the food that is consumed from the container. This is one of the prime pathways for EDCs to enter the food chain. Apart from entering the food chain, EDCs have also been found to be present in surface water. It is a known fact that EDCs, such as bisphenol S (BPS), is being used as an alternative to bisphenol A in personal care products, textiles, thermal papers, and phenolic resins [48]. BPS enters the water stream mainly due to the dumping of these materials into water bodies. BPS leaches out from these materials into the water bodies. In water bodies, BPS has been commonly detected at levels between 10 ng/L and 300 μg/L [49]. Although it has been detected in small amounts, its adverse effects are devastating. BPS has the potential to alter the function of the human endocrine system. In addition to BPS, other EDCs have also found in water streams in recent years. 

EDCs have been found to interrupt insulin secretion, as well as glucocorticoid, estrogenic, and thyroid hormone pathways. This results in fetal growth retardation, thyroid dysfunction in both the mother and the fetus, and neurological disorders [50]. Toxicology reports also suggest that gene mutation might occur with exposure to EDCs in the human body [51]. Thus, researchers have actively explored ways to remove EDCs from water sources and to eventually prevent them from entering the human food chain. 

Several techniques have been tried and tested for the removal of EDCs from water sources. Techniques, such as the Fenton process, oxidation process, photocatalytic degradation, membrane filtration, adsorption, and microalgae, have been used to remove EDCs, with various success rates. Though some methods, like the Fenton process, demonstrated close to 100% removal of EDCs, the scalability of such techniques is questionable [52]. The overall cost of using the Fenton process is relatively larger compared to other techniques, such as adsorption and microalgae removal. Thus, finding the most appropriate technique which balances overall cost and removal efficiency is key. Considering such a scenario, the usage of aerobic granules in the removal of EDCs can be explored. The overall cost of operating the aerobic granular technology was found to be relatively cheaper compared to other techniques, such as membrane systems.

### 3.3. Microplastics

Microplastics are tiny fragments of plastic of less than 5 mm in size which have become an environmental concern in recent years. In general, the usage of plastic has penetrated deep into our daily life. Almost all aspects of a human life are centered around plastic in daily life. Its low cost, durability, flexibility, and corrosion resistance have made plasticubiquitous [53]. Almost 50% of plastic usage involves single-use plastics [54]. Plastics are not 100% biodegradable, but they fragment upon long exposure to sunlight and bacterial activity (upon landfilling). The fragmentation of plastics forms microplastics in the environment [55]. Microplastics are defined as plastics with sizes less than 5 mm. 

Microplastics are further categorized into smaller size fractions, including nanoplastics (<1 μm), which have been increasingly reported in recent times. Though they are often grouped under the category of microplastics, nanoplastics are of particular concern, as they can be harmful to living beings. They can penetrate biological membranes, accumulate in tissues, and potentially cause severe toxicological effects. 

Microplastics can be generated directly and indirectly. Direct generation of microplastics originates from the release of microbeads from cosmetics, synthetic textiles, tires, plastic pellets and road markings [56]. On the other hand, the indirect release of microplastics into the environment originates from the biodegradation of plastics into micro-sized plastics [57]. The combination of the direct and indirect release of microplastics has resulted in a surge in the amount of microplastics in the environment over the past 10 years. 

Microplastics are generated on land, but they end up on land, in the ocean, and in the air. In municipal wastewater, on average, the amount of microplastics detected is around 124.04 pcs MP/L [58]. Microplastics are light and less dense, which enable them to be present in various environment [59]. To make things worse, the microplastics end up in our food chain. The microplastics present in the ocean have been mistakenly consumed by fish as food. In aquatic lifeforms, the consumption of microplastics has resulted in gill inflammation, liver damage, kidney damage, and reproduction disruption [60]. On the other hand, the microplastics found in fish are later transported into the human body upon the consumption of these fish. Due to the consumption of these microplastics, the physiological aspect of the human body changes over time. The immune system, hormonal balance, reproductive systems, and other aspects of human wellbeing are affected by the ingestion of microplastics [61]. Microplastics could be the one of the prime factors for cancer in the future if their presence is left untreated. 

To solve the issue of microplastics, an effective removal system is needed. As of now, existing technologies are unable to remove microplastics from the environment, be it from the air or from surface water. To date, the adsorption technique has been found to remove some microplastics from the environment [62,63,64]. Meanwhile, other primary treatment techniques, such as coagulation and flocculation, dissolved air floatation, and sedimentation, have not produced effective removal of microplastics from water sources [65]. Therefore, an effective treatment system has been actively researched for the past 10 years. 

Treatment methods, such as membrane bioreactor technology, sand filtration, electrocoagulation, biochar filters, ozonation, and aerobic granular technology, are some of the tried and tested techniques to remove microplastics from the water. The efficiency of these techniques varies from 75 to 100% removal of microplastics from water sources [66]. Though some techniques produced 100% microplastic removal, the cost involved in these techniques was huge and not sustainable in the long run. It has been reported that *Comamonas acidovorans* managed to completely degrade the microplastics in water sources [67]. However, this pure culture removal technique is costly and susceptible to toxicity contamination. On contrary, aerobic granular technology, which is a mixed culture, has exhibited removal rates between 77 to 94%, which is slightly lower than the pure culture [68]. However, the operational cost of aerobic granular technology is more economical compared to pure culture reactors [69]. Thus, a balance between treatment efficiency and cost effectiveness must be found to make microplastic removal from water sources sustainable.

### 3.4. Per- and Polyfluoroalkyl Substances (PFASs)

Per- and polyfluoroalkyl substances (PFASs) have been used in daily life since 1950s. Initially, PFASs were employed as a component in fire-extinguishing foams. Over the next half-century, PFAS application expanded significantly, and today PFASs are found in a wide range of consumer products. Generally, PFASs are known for their unique properties, including heat resistance, hydrophobicity, chemical resistance, lipophobicity, and low friction coefficient. 

PFASs are commonly present in household items, such as non-stick pans, fire extinguishers, water-resistant jackets, electronics, cosmetics, shampoo, paints, pesticides, fast food packaging, and batteries. The carbon–fluorine bond is one of the strongest in organic chemistry, making these compounds extremely stable and resistant to degradation [70]. Usually, PFASs are manufactured using electrochemical fluorination and telomerization processes. 

Due to their stability and versatility, PFASs have been widely used across industries and other aspects of our daily life. Unfortunately, PFASs have been labeled as harmful to human beings, even at very low concentrations [71]. It is worth noting that the limits of some PFASs, such asperfluorooctanoic acid (PFOA) and Perfluorooctane Sulfonate (PFOS), in drinking water were set at 4.0 ppt in 2024, following ongoing research into their adverse health effects [72]. 

In addition to that, certain PFASs, such as PFOA and PFOS, have been categorized as a persistent organic pollutant under the Stockholm Convention, which was adopted in 2001 [73]. The major sources of PFASs were found to be industrial applications, consumer products, wastewater treatment plants, and landfill leachate. 

**Figure 1:**
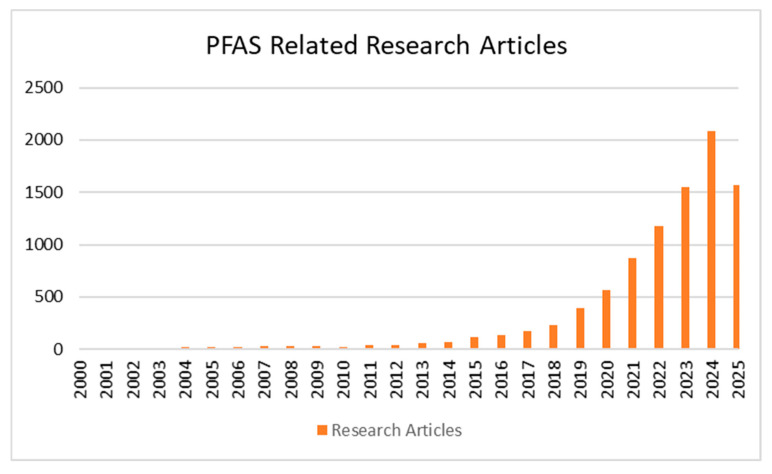
Growth in research articles related to PFASs (data extracted from Scopus).

The adverse impact of PFASs on human health has been reported extensively. It has been reported that PFASs have resulted in increased cancer risk, delayed the onset of puberty among girls, caused bone mineral density reduction, reduced the ability of the immune system, caused metabolic alterations, and resulted in an increased risk of diabetes. All these health risks have increased the intensity of the research related to PFASs. Figure 1 illustrates the significant growth in the number of the PFAS-related research articles published in Scopus over the past 25 years. The increasing trend of research papers published which are related to PFASs is evident. These research papers mainly focus on effective methods to remove PFASs from water sources, including surface water and wastewater, which have been identified as prime sources of PFASs which enter the human body. 

The concentration of PFASs detected in the municipal wastewater was between 24.1 and 66.9 μg/L [74]. In water bodies, the presence of PFASs is noted in the runoff from fire-extinguishing foam, textile industries, and debris from construction sites. When these water sources are used for drinking, PFASs bioaccumulate in the human body if no proper filtration is used. In addition to water sources, PFASs have been found in certain edible crops, likely due to the usage of contaminated water and pesticides during their growth. 

To address this issue, various techniques have been developed to remove PFASs from water sources. Among the techniques that have been widely implemented are adsorption, plasma treatment, foam fractionation, photocatalysis, and electrochemical remediation [75,76,77]. The removal efficiency of these techniques varies from 70 to 99%. 

The differences in removal efficiency are largely due to the nature of the removal techniques. Some techniques, such as adsorption, transfer PFASs from one medium to the other medium without mineralizing them. In such techniques, the efficiency is conventionally lower than 95%. Meanwhile, in techniques where the PFASs are mineralized into harmless byproducts, the removal efficiency is usually reported as being above 99%. However, techniques which result in mineralization of PFASs are usually costly due to their energy-intensive natures. 

## 4. Reactor Configuration to Cultivate Aerobic Granules

### 4.1. Sequencing Batch Reactor (SBR)

Aerobic granules are typically cultivated in sequencing batch reactors (SBRs), which operate in a cyclic mode throughout the day. A complete cycle of an SBR reaction consists of four or five phases, namely feeding, reaction, settling, decanting, and idle phases (optional). The duration of each phase may vary with the operational conditions, while the total cycle time ranges between 4 and 12 h [78,79]. 

The feeding phase is usually kept shorter compared to the other phases. During this phase, fresh substrate or wastewater is fed into the SBR. Meanwhile, the reaction phase is generally the longest phase in a cycle, during which the degradation of the substrate occurs. For example, in a 6 h cycle, the feeding phase normally ranges between 10 and 20 min, followed by the reaction phase (averaging around 324 to 340 min), with 0.5 to 10 min of settling and 6 to 9.5 min of decanting [79,80,81].

Establishing a feast–famine feeding strategy during the initial phases is crucial for the aerobic granules’ formation. The feast phase refers to the period when the external substrates are available for microbial uptake, typically occurring during the feeding phase and possibly extending to the early part of the reaction phase [82]. Once the external substrate has been fully consumed, the reaction enters the famine phase, in which the microbes rely on internal storage compounds for maintenance. For the aerobic granules to survive during the famine period, they store polyhydroxyalkanoate (PHA) during the feast period, which is later consumed as an internal carbon and energy source. The establishment of a distinct feast–famine period in the SBR is crucial for the successful cultivation of aerobic granules.

On the other hand, the height over diameter (H/D) ratio of the SBR should, ideally, be in the range from 5 to 10 [79,83]. By having this ratio, it imposes higher hydrodynamic shear force on the activated sludge and on the developing aerobic granules. Increased hydrodynamic shear force increases the stress condition on the microorganisms, which, in turn, increases the secretion of extracellular polymeric substance (EPSs). This enhances cell-to-cell adhesion, facilitating the formation and growth of granules. EPS secretion also promotes the adhesion of new cells onto the existing granules, contributing to granule maturation.

Furthermore, the SBR is also subjected to selection pressure through a gradual reduction in the settling time from the day of inoculation. This strategy promotes the selection of dense and compact granules in the SBR. In a typical lab-scale SBR setup, the settling time is initially set to around 10 min upon inoculation. As the SBR operation progresses and granulation matures, the settling time is reduced gradually to 1.5 min [79]. Due to this settling selection pressure, only the granules which settle within 1.5 min remain in the reactor, while the poorly settling flocs are washed out from the SBR. Settling pressure not only promotes compact granule formation but also improves the biomass–effluent separation. 

In other words, the need to use a clarifier could be eliminated due to the good separation between the aerobic granules and treated wastewater. The supernatant after the settling phase contains minimal biomass residues, indicating efficient solid–liquid separation, which is driven by the granules’ characteristics and the SBR operational strategy. 

Nevertheless, the usage of SBR is limited by the volume of each batch of wastewater that can be treated. As the SBR operates in a cyclic mode, the incoming wastewater can be fed to the SBR only after the completion of each cycle. This requires SBRs with the capacity to treat large volumes of wastewater in each cycle of operation. Naturally, this will incur an additional cost due to the large reactor volume required for sectors producing high wastewater loads. As a result, the continuous flow system has been adopted as an alternative in recent years.

### 4.2. Continuous Flow System

The continuous flow system for the development and application of aerobic granules is regarded as an advancement over the conventional batch reactor. One of its key advantages is the ability to retrofit existing wastewater treatment plants with this technology.

Various configurations of continuous flow reactors have been developed to cultivate aerobic granules. Rosa-Masegosa, et al. [84] reported that a single-chamber continuous flow bioreactor was able to produce the aerobic granules from sewage wastewater across a range of organic loading rates, between 0.45 and 1.85 kg COD/m^3^ day. The developed granules achieved more than 80% COD removal efficiency. Furthermore, an increase in OLR led to larger granule sizes. Microbial analysis revealed a diverse microbial community within the developed granules. 

Additionally, a vertical continuous flow reactor was applied at pilot-scale to treat rural domestic wastewater [85]. The developed aerobic granules were found to remove the COD, ammonia nitrogen, total nitrogen, and total phosphorus at rates of 90%, 94%, 75%, and 93%, respectively. These results indicate that the aerobic granules developed using the continuous flow reactor are as fully functional as the ones developed in the batch reactor. 

Meanwhile, Franchi, et al. [86] studied continuous flow reactors designed with both tanks-in-series and plug flow reactors. Aerobic granules were successfully cultivated in both configurations. The organic loading rate (OLR) and the upflow velocity were investigated, and it was observed that granule size increased with OLR (ranging from 0.7 to 4.1 kg COD/m^3^.day). Moreover, the increase in the F/M ratio enhanced the microbial biodiversity of the granules.

Furthermore, the developed aerobic granules appear to function well for the nitritation process as well. Previously, it was reported that granules were not able to complete nitritation at low temperatures. Contrary to the conventional microorganisms, the developed aerobic granules using a continuous flow reactor were reported to achieve stable nitritation at low temperature (5 °C), in which the nitrite production was 0.29 kg/m^3^.day [87]. It was also reported that the granules tended to reduce in size upon a decrease in temperature, and that there was a reduction in calcium content during the temperature decrease.

Apart from this, rapid granulation of aerobic granules in a continuous flow membrane reactor used to treat the municipal wastewater was found by Yang, et al. [88]. In this research, it was found that elevated Ca^2+^ coupled with an increase in extracellular polymeric substances (EPSs) promoted the quick formation of aerobic granules in the continuous flow reactor. The formed aerobic granules were notable for their functional and structural stability throughout the operation.

These studies prove that a continuous flow reactor can be successfully used to develop aerobic granules and to deploy them for the degradation of wastewater. The promising outcomes coupled with the flexibility to retrofit the existing wastewater treatment facilities, along with promising performance outcomes, makes the continuous flow treatment system a compelling option for a large-scale wastewater treatment application.

However, the development of aerobic granules in wastewater containing ECs remains insufficiently explored. The potential inhibitory effects of ECs on microbial activity during aerobic granule formation remains a largely unexplored territory. Though the role of the EPSs in the granules’ stability has been discussed extensively, the effect of ECs on EPS secretion is still not well understood. Furthermore, comparative assessments of the cost-effectiveness of continuous flow reactors versus conventional batch reactors, particularly in the context of EC removal, are scarce, and warrant further investigation. 

## 5. Emerging Contaminant Removal Mechanism

The removal mechanism of ECs from water sources needs to be thoroughly investigated to understand and potentially improve the removal efficiency [89]. One of the key findings in EC removal using aerobic granules is the removal efficiency variation. For stable EC removal using aerobic granules, the variation in removal efficiency should be minimized. To minimize the removal variation, the mechanism of the EC removal in aerobic granules has to be comprehended. 

**Figure 2:**
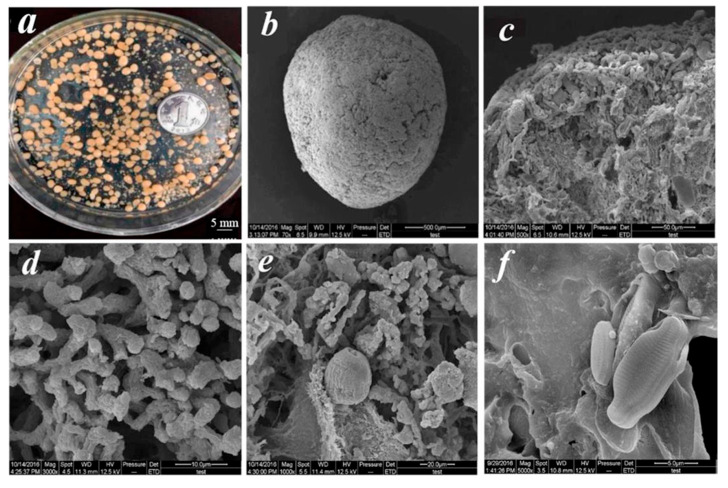
Digital and scanning electron microscopic (SEM) images of mature aerobic granule samples at steady state. (**a**) Digital image of the morphology of mature aerobic granules in a phototrophic state. Scanning electron microscopic (SEM) images of (**b**) an entire aerobic granule in a phototrophic state; (**c**–**f**) the outer surface of the phototrophic granule reproduced from [94]. Reprinted with permission from Elsevier [94].

Aerobic granules function as effective adsorbents due to the intrinsic physicochemical properties. It has been well documented that aerobic granules have a high surface area, are highly porous, and are enriched with functional groups [90,91]. The cross-section of the aerobic granules observed under a scanning electron microscope (SEM) confirms that the aerobic granules are porous (Figure 2). It is worth noting that in aerobic granules, both physisorption and chemisorption have been reported. As the aerobic granules are rich with functional groups, these types of adsorptions are viable in the aerobic granules. The functional groups that have been found on the aerobic granules are carboxyl, amino, and hydroxyl groups [92,93]. Apparently, aerobic granules are enriched with functional groups due to the presence of extracellular polymeric substances (EPSs). In aerobic granules, there are three types of EPSs, namely loosely bound EPSs, tightly bound EPSs, and soluble microbial products [92]. All these types of EPSs contain the aforementioned functional groups and contribute towards the adsorption process. 

Bodle and Kirkland [95] reported that the aerobic granules were used to remove an abiotic pharmaceutical mixture containing diclofenac, erythromycin, and gemfibrozil. It was reported that the sorption sites of the aerobic granules were not completely occupied, although the concentration of the mixture was 150 μg/L. Meanwhile, Zheng, et al. [96] studied the adsorption of 17β-estradiol (E2) and 17α-ethinylestradiol (EE2) using aerobic granules. It was reported that the EE2 removal was found to be adsorption-based, with a removal efficiency of 84.8%. The adsorption process for EE2 was found to be due to heterogeneous multilayers and chemisorption. The functional groups of the enriched aerobic granules were accredited for the adsorption process of EE2.

On the other hand, inactive aerobic granules have also been used for the removal of pharmaceutical products. In the work of Burzio, et al. [97], biologically inactive granules were used to remove micropollutants (sertraline, citalopram, clarithromycin, erythromycin, levonorgestrel, estradiol, ethinylestradiol, ketoconazole, and losartan) with various charges. These micropollutants could be removed using the biologically inactive granules. This phenomenon indicates that the removal process in the aerobic granules was dependent on adsorption rather than biodegradation. 

In addition to the adsorption process, the biodegradation of contaminants has also been reported in aerobic granules. In earlier works on dyes, the degradation of aromatic amine compounds through biodegradation has been reported. Upon mineralization of the intermediate products, the final products of the biodegradation are CO_2_, H_2_O, and N_2_. Similar mechanisms have been reported for emerging contaminants. However, some emerging contaminants, such as PFAS, are recalcitrant towards the biodegradation mechanism. The strong bond of C-F in the compounds make the biodegradation of PFASs difficult. The biodegradation mechanism has been discovered to be the reason for the mineralization of EDCs, such as estrogens and estradiols. These compounds are relatively easier to biodegrade compared to other ECs. 

Based on the reported works, it is apparent that adsorption and biodegradation are the predominant mechanisms for removing ECs using aerobic granules. However, the adsorption mechanism appears to be slightly more dominating in terms of removing ECs when using aerobic granules compared to biodegradation for some ECs. Certain complex organic compounds, such as carbamazepine and bisphenol A, exhibit limited biodegradability under aerobic conditions, even within aerobic granules. Furthermore, the hydrophobic nature of ECs promotes interaction with hydrophobic groups on the surface of aerobic granules. Additionally, the presence of functional groups, such as hydroxyl and amino, facilitates hydrogen bonding between ECs and functional groups on the aerobic granules. On the other hand, the biodegradation processes of ECs in the aerobic granules are largely dependent on the compound structure, environmental conditions, and microbial community. As some EC compounds are complex, the biodegradation process is a scarce phenomenon.

However, some EC compounds undergo degradation via specific pathways. The microbial community within the matrix of aerobic granules facilitates this biodegradation process. EC compounds, such as estrogen, undergo hydroxylation, followed by ring cleavage for complete mineralization [98]. This process is usually mediated by microorganisms, such as *Sphingomonas* and *Novosphingomium* spp. On the other hand, diclofenac was found to be degraded until complete mineralization through the use of *Comamonas* and *Pseudomonas* spp. It undergoes hydroxylation and decarboxylation during the degradation process [99]. Meanwhile, erythromycin and some macrolides undergo demethylation and hydrolysis during the degradation process, which is usually mediated by microorganisms, such as *Bacillus* spp [100]. At the same time, some stable compounds, such as carbamazepine and bisphenol A, require much rigorous pathways, such as co-metabolic pathways, for their partial degradation [101].

## 6. Performance Evaluation

### 6.1. Removal Efficiency

The removal efficiency of ECs serves as a benchmark to gauge the suitability of the aerobic granules for the purpose of EC removal. Table 2 summarizes the reported removal efficiencies of various ECs by aerobic granules. The performance varies depending on the physicochemical properties of the contaminants and characteristics of the granules.

These results indicate that aerobic granules are capable of achieving high removal efficiency for many ECs. The efficacy of the aerobic granules in removing ECs has been proven in both synthetic and real municipal wastewater. In the work performed by Burzio, et al. [107] and Balest, et al. [108], in which real municipal wastewater was used, the removal efficiency for most ECs exceeded 60%, while certain compounds, such as ibuprofen, exhibited removal rates as high as 98.3%. However, a significant limitation is the potential inhibitory effect of certain contaminants on the microbial community within the granules. As these contaminants have a high possibility of inhibiting microorganisms, the removal capability of the aerobic granules could be affected.

In the work of Tang, et al. [111], it was reported that ibuprofen was found to reduce EPS secretion, impair the settling ability of the granules, and alter the microbial community composition in the aerobic granules. Similarly, Gan, et al. [112] found that polystyrene microplastics inhibit the specific nitrite reduction rate (SNIRR) and specific nitrate reduction rate (SNRR) at a polystyrene microplastic concentration of 100 mg/L. Meanwhile, Ilieva, et al. [102] also noted that the PFAS exposure caused the inhibition of microorganisms in aerobic granules. It was reported that the biomass concentration and settling ability exhibited declining trends with the exposure of PFASs to the aerobic granules.

To mitigate such inhibitory effects, researchers have introduced acclimatization strategies. In the work of Ilieva, et al. [102], the aerobic granules were acclimatized to the PFASs before the removal process took place. It was found that the acclimatized aerobic granules were able to retain their biomass concentration and settling ability in contrast to the control reactor, which was not acclimatized. However, it is important to note that acclimatization does not guarantee complete degradation for all ECs. Some recalcitrant compounds, such as carbamazepine and naproxen, were not effectively degraded by the aerobic granules even after an acclimatization period of 30 days [113].

### 6.2. Granule Morphology and Stability

Aerobic granules’ performance in removing the emerging contaminants is largely dependent on the structure of the granules. The cross-section of the aerobic granules reveals the layered structure surrounding the core. The outermost layer is the aerobic zone, the middle layer is the anoxic zone, and the core of the granules is the anaerobic zone. The mechanisms of contaminant removal in each layer are different from one another, as the basic reactions are different. For example, the presence of oxygen in the aerobic zone degrades the contaminants via the oxidation process. He, et al. [114] reported that tetracycline, which is a type of antibiotic, was removed from wastewater using manganese oxidizing aerobic granular sludge via an oxidation process, which means that it was removed by the aerobic zone of the aerobic granules. 

As the aerobic granules are made of a multilayered structure, the removal of emerging contaminants sometimes involves two different layers. Some emerging contaminants, such as gemfibrozil, were found to be completely removed when using the aerobic granules. The removal of gemfibrozil was aided by the presence of various types of bacteria at different strata of the aerobic granules. The coexistence of *J111*, *Xanthomonadaceae*, *OLB5*, and *Weeksellaceae* was found to have degraded the gemfibrozil in the aerobic granules [95]. On the other hand, 17α-ethinylestradiol (EE2) and 4-nonylphenol (NP) were also found to be degraded by the aerobic granules. Uniquely, it was reported that, at the initial stages, the removal of EE2 and NPs were adsorption-dominated. However, as the aerobic granules became saturated, the biodegradation of these contaminants took place [115].

In addition to the layered structure of the aerobic granules, it is also enriched with extracellular polymeric substances. The EPSs are categorized as loosely bound EPS, tightly bound EPS, and soluble microbial particles [116]. All these categories of EPS are enriched with functional groups which also aid in the adsorption process. Functional groups, such as carbonyl, phosphate, and amine, aids the removal process of the emerging contaminants using aerobic granules. Zhao, et al. [117] reported that the EPS can bind with antibiotics in several ways, namely surface complexation, hydrophobic interaction, electron donor acceptor, and hydrophobic bridging. As the EPS is enriched with diverse functional groups, the antibiotics tends to bond with these functional groups in various ways. The interaction makes the removal process of antibiotics possible in aerobic granules. 

Although the characteristics of the aerobic granules, such as their multilayer structure, and the EPSs have facilitated the removal of emerging contaminants, these contaminants can also inhibit the activity of the microorganisms in aerobic granules or become toxic shocks to the microorganisms in aerobic granules. As a result, this would reduce the removal efficiency of the emerging contaminants when using aerobic granules. Guo, et al. [118] reported that emerging contaminants have exhibited biotoxicity, bioaccumulation, and environment persistence. In the study conducted by Zhu, et al. [119], it was found that the 4-chloroaniline can cause toxicity to aerobic granules at 200 mg/L. The aerobic granules were found to be disintegrated by the presence of 4-chloroaniline. Because of the disintegration, the performance of the aerobic granules in removing the contaminants was hindered. Similarly, perfluorooctanoic acid (PFOA) has been found to hinder the performance of the aerobic granules as well. In the study of Xie, et al. [120], the formation of the aerobic granules was found to be delayed in the presence of 0.1 mg/L of PFOA. At the maturation stage of the aerobic granules, the proportion of large aerobic granules was found to be relatively lower. Furthermore, the presence of PFOA also led to a decline in the nutrient removal process, in addition to the changes in the microbial community in aerobic granules. Likewise, the presence of carbamazepine has been reported to have interrupted the granules’ formation. The carbamazepine affected the mechanism of the granules’ formation, which led to a reduction in the granules’ size [115]. 

## 7. Challenges and Future Direction

### 7.1. Scalability and Operational Challenges

At lab-scale, the application of aerobic granules to remove emerging contaminants appear to be attractive. Apart from the superior capability of aerobic granules in removing emerging contaminants, the aerobic granular system occupies only about 25 to 50% of the conventional activated sludge system. Additionally, the energy requirement for the operation of the aerobic granular system is relatively lower compared to the conventional activated sludge system. Furthermore, the aerobic granules were better able to sustain the toxic environment when compared to the conventional activated sludge. All these characteristics make the aerobic granules a better option compared to the existing activated sludge system.

Nevertheless, there are challenges that remain unresolved for the upscaling of this technology to the industrial-scale. To date, the Nereda^®^ wastewater treatment plants in the Netherlands, United Kingdom, Brazil, United States, Australia, Portugal, France, and Canada have successfully implemented aerobic granules to treat the wastewater [121]. However, this method’s expansion to worldwide usage has been hampered by a few factors. One of the primary factors for the limited usage of aerobic granular technology is the slow granulation process for low-strength wastewater. Apparently, the low amount of substrate loading and limited nutrient availability in the low-strength wastewater has slowed down the granulation process. Due to the low availability of the substrate, the amount of EPS released has also been reported to be low [122]. As such, the granulation initiation process had been delayed due to the low amount of EPS, which acts as the granulation precursor. Thus, co-substrates have to be added to the low-strength wastewater to enhance the granulation process [123]. 

Furthermore, the long-term instability of the aerobic granules remains as a challenge for wastewater operation. One of the key components in the aerobic granulation process is the presence of filamentous bacteria at the initial stages of the granulation. It provides the backbone for granulation to take place. However, overgrowth of filamentous bacteria has been reported to have led to granule instability and, eventually, their disintegration. 

Besides that, the growth of aerobic granules also poses a challenge for the instability cause. The continuous growth in terms of granule size makes the inner core of the aerobic granules limited for mass transfer. As such, the core of the aerobic granules becomes weaker as the diameter of the aerobic granules grows. Due to the limited organic compound at the core because of the mass transfer limitation, the EPS is consumed as the substrate. The EPS which forms the backbone of the granules’ structure stability becomes weaker which eventually leads to the disintegration. 

It has been discussed earlier that continuous flow reactors are the way forward for the implementation of aerobic granular technology at a large scale. They allow this technology to be used at a large scale. However, several characteristics of the continuous flow reactor makes aerobic granular technology implementation complex. When conventionally developed in a batch reactor, the feast–famine stress condition is imposed on the activated sludge, which stimulates the secretion of the EPS. However, this stress condition is challenging to replicate in the continuous flow reactor due to the variation in the influent. The substrate concentration gradient was found to be too low to create the feast–famine condition in the continuous flow reactor. 

Additionally, the shear stress imposed during the granulation process is different in lab-scale and industrial-scale reactors. In lab-scale, a column reactor is used, which imposes shear stress on the activated sludge. which eventually turns into aerobic granules. On the other hand, for the continuous flow reactor, a similar shear stress could not be imposed due to the configuration of the reactor. The continuous flow reactor normally has a flat geometry, which imposes low shear stress. As such, the granulation process in continuous flow reactors is relatively slower. 

Moreover, the microbial community diversity in aerobic granules poses another challenge for this system to operate. Due to the influent variation, the aerobic granules exhibit shifting in the microbial community. It is crucial to ensure that certain microbial communities remain in the aerobic granules for them to remove emerging contaminants. However, a combination of both varying influent and inhibition of the microorganisms by the influent has been reported to shift the microbial community in the aerobic granules. This leads to the failure of the aerobic granules in treating the emerging contaminants. In fact, the changes in pH have also been found to have a negative effect towards the microbial community.

### 7.2. Integration with Other Technologies

The aerobic granular system has been reported as being integrated with other technologies in order efficiently to remove ECs. As described in the earlier section, the removal rates of ECs in some of the studies were barely 50% or lower. In such cases, the recalcitrant ECs has been the cause. Therefore, aerobic granular technology has been integrated with other technologies to improve overall EC removal from wastewater. Completely or partially biodegradable ECs can be removed by aerobic granules, while the recalcitrant ECs can be removed in the subsequent process.

Aerobic granular technology has been integrated with advanced oxidation process. In this process, the advanced oxidation process was integrated pre- or post-aerobic granular technology usage. Advance oxidation processes, such as the Fenton process, ozonation, and UV/H_2_O_2_, have been employed together with aerobic granular technology to remove ECs. The advanced oxidation processes mainly focus on producing hydroxyl radicals to oxidize pharmaceuticals and endocrine-disrupting compounds. Through this integration the overall removal rate of pharmaceuticals and EDCs reached over 90%.

On the other hand, aerobic granular technology has also been integrated with membrane bioreactors. Principally, the membrane bioreactor removes ECs through physical methods, while aerobic granules biodegrade the ECs. Conventionally, the membrane bioreactor is applied during post-aerobic granular technology treatment. It has been reported that pharmaceutical components are degraded by aerobic granules, while the membrane bioreactor removes the recalcitrant ECs via physical means. 

Aerobic granular technology has also been integrated with activated carbon to improve the overall EC removal efficiency. This integration is performed in such a way that the activated carbon and aerobic granules coexist. Through this coexistence, the ECs that are present in wastewater are attached to the activated carbon through the adsorption process. The adsorption process concentrates the ECs on the activated carbon, which can be biodegraded by the aerobic granules. This integrated system mostly works well with pharmaceutical products, which can be biodegraded by the aerobic granules. Other products, like bisphenol A, have also been found to be effectively removed using this technique.

Though aerobic granules themselves have the potential to remove a wide spectrum of ECs, their integration with other technologies enhances their performance to better remove ECs. Integrated systems offer complementary mechanisms for the removal of ECs pre- or post-aerobic granular treatment systems. This approach allows a wider spectrum of ECs to be removed from wastewater. 

### 7.3. Current Legislation on Emerging Contaminants

Emerging contaminants, including PPCPs, EDCs, microplastics, and PFASs, are not comprehensively regulated globally. However, in recent years, legislation on emerging contaminants is evolving towards identifying new synthetic substances which could potentially harm human and biota, setting their discharge concentration, and determining their acute and chronic toxicity levels. Notably, the 2019 Basel Convention Plastic Waste Amendments specifically created a framework on the transboundary movement of plastic waste, including plastic waste that contains emerging contaminants [124]. This framework strengthens international control of plastic waste and will, eventually, facilitate environmental damage control. In a similar vein, the European Union, through its Water Framework Directive (2000/60/EC) and ‘Watchlist’ mechanism, has required its members to monitor certain priority emerging contaminants, such as hormones and diclofenac [125,126]. In the Asian region, countries, like Japan and China, have undertaken initiatives to monitor and introduce discharge limit for contaminants, such as PFASs and pharmaceutical products [127,128]. Nevertheless, a unified global standard for the emerging contaminants is critically warranted, given the potential risks posed by emerging contaminants to human beings and the environment. The standard should define the allowable limits across multiple environmental media, especially for water and air.

## 8. Conclusions

Aerobic granular sludge has exhibited significant potential for the removal of ECs from municipal and industrial wastewater. The high removal efficiency (> 85%) exhibited by aerobic granules for PPCPs, PFASs, EDCs. and microplastics shows the true potential of aerobic granules for these applications. The stratified structure of the aerobic granules enables multiple removal mechanisms, while the coexistence of EPSs surrounding the aerobic granules further enhances the EC removal through biodegradation and adsorption. It has been identified that operational parameters, such as the duration of phases, H/D ratio, and settling time, play significant roles in the operation of aerobic granular technology. However, the transformation of this technology from lab-scale work to industrial-scale deployment remains a challenge due to issues including granule stability, continuous flow operation, and the lack of a comprehensive understanding of EC effects on the microbial activities of aerobic granules. To fully harness the potential of this technology, future work should focus on comprehensive long-term pilot plant studies and techno-economic analysis.

## Figures and Tables

**Table 1:** Comparison of activated sludge and aerobic granules.

Characteristics	Activated Sludge	Aerobic Granules	References
Compactness	Low compact	Highly compact	[20]
Structure	Single layer	Multilayer	[21]
Settling ability	Low settling ability	High settling ability	[22]
Robustness	Less robust	Highly robust	[6]
Footprint	Large footprint	Small footprint	[23]
Sludge volume index (SVI)	High SVI	Low SVI	[24]
Resistance to shock loading	Low resistance	High resistance	[25]
Energy efficiency	High energy needed	Low energy	[11]
Treatment efficiency	Requires multiple stages	Simultaneous removal of COD, nitrogen, and phosphorus	[26]
Biomass retention	Low retention of biomass	High retention of biomass due to good settling	[8]

**Table 2:** Removal efficiency of ECs by aerobic granules.

Contaminant	Removal Efficiency	References
Gemfibrozil Diclofenac Erythromycin	10–100%	[96]
PFASs	17–100%	[102]
Venlafaxine (VNF) Tramadol	89%	[103]
Carbamazepine	60–85%	[104]
Ketoprofen	50–60%	
Cyclophosphamide	70%	
Trimethoprim	70%	
Kanamycin tetracycline Ciprofloxacin ampicillin Erythromycin	88.4%	[105]
Sulfamethoxazole	99%	[106]
Tetracyclin	79.17%	[5]
Sulfamethoxazole	70.86%	
Ofloxacin	25.73%	
Roxithromycin	88.93%	
Paracetamol	98.1%	[107]
Ibuprofen	98.3%	
Bisphenol A	95.6%	
Ciprofloxacin	65.8%	
17β-estradiol	93.5%	[97]
17α-ethinylestradiol	84.8%	
Estrone 17β-estradiol 17α-ethynylestradiol Bisphenol A 4-*tert*-octylphenol	60% 69% 93% 81%	[108]
Microplastics	74–95%	[109]
PFOS	>85%	[110]
